# The Institutional Library

**Published:** 1917-11-03

**Authors:** 


### THE INSTITUTIONAL LIBRARY.

**The Small Community Hospital.** A Series of Eight Papers by John Allan Hornby, M.D., Editor of *The Modern Hospital.* Reprinted from that paper. (St. Louis. 1917.)

This book contains the case for the cottage hospital in America. That case rests upon the premiss that less than 12 per cent. of the sick in America use the hospitals; the remainder, it seems, either is not treated or is treated insufficiently at home. It is argued that every two or three thousand people require a hospital of some sort, and to show how nearly the English cottage hospital plan is the type, we need only add that reconstructed private houses or factories are suggested as being better than no hospital at all. The original Axminster Cottage Hospital, which only moved into a new building about five years ago, was, by the way, the factory at which the famous Axminster carpets were first made before the business moved to the Midlands. The method by which such hospitals can be created among the "small communities" of America is then discussed. Ways and means, construction, equipment, organisation, staffing, design, and maintenance are then dealt with in succeeding papers. The cottage hospital here is such an established institution, and its range from four beds in a cottage to a specially designed institution of sixteen to twenty beds is so wide, that this book cannot fail to make stimulating reading. For the old, if they have learnt wisdom as well as golf, have almost as much to learn from the young as the young have from the old. So this proposal to establish in America an institution similar to a traditional institution here is at once a stimulus and a criticism. While it makes us realise the value we have, it suggests points which we ought to ponder. The need is sufficiently like our own to create sympathy: the conditions are sufficiently different to make us think. We are glad that the papers have been reprinted, but we believe that our contemporary is sufficiently read in this country to make a recapitulation of the contents of each article an unnecessary office to the author. Every cottage hospital chairman and secretary should read the book, and all, indeed, who desire a critical knowledge of one of the most valuable of English institutions.

**The Treatment of War Wounds.** By W. W. Keen, M.D., LL.D. Illustrated. (Philadelphia and London: W. B. Saunders and Co. 1917. 8vo. Pp. 169.)

This report has been compiled at the request of the National Research Council of the United States, and Professor Keen states modestly that it is only a memorandum on some of the more important and most recent advances in the treatment of war wounds. It is, however, very much more than a memorandum, for it is a clearly written account by a master of surgery which enables the reader to obtain a sound knowledge of war surgery. The gift of popularising scientific knowledge is very rare, and is only possessed by the greatest teachers. The gift was given to Faraday, to Tyndall, to Huxley, and to Michael Foster. Professor Keen in this book shows that he, too, has it in abundance. Born in 1837, his memory easily recalls the American War, and he states that "personally I never saw a single case of gas infection in the Civil War." In the course of his long life he has seen so many changes of surgical opinion that his conclusions may be relied upon, although the evidence upon which he bases them is due to the testimony of others. The value of the book lies in the help it gives to those who have not yet had experience in military surgery, for it teaches what has already been tried and approved, and thus gives a starting-point from which still farther advances may be made. The Dakin-Carrel method as it is carried out at Compiègne and La Panne is explained in detail with ample illustrations. There is a short chapter upon the value of stereo-fluoroscopy in the localisation of foreign bodies previous to their extraction. The memorandum on tetanus issued by the British War Office is given in full, and the work done at the Rockefeller Institute receives adequate recognition. The conclusions arrived at by Professor Harvey Cushing in connection with head injuries are quoted with the approval they deserve. An interesting example is given of the urgency of war surgery from the experience of Lockwood and his colleagues, who, "after being rushed with the wounded from four days of continuous and active fighting, suddenly in one night, between 9 and 12 o'clock, received ninety-six operative cases—one every two minutes. Of these thirty-six were abdominal cases, and of these thirty-two were operated upon. One significant statement is that the surgeon was usually obliged to operate alone."

So rapid has been the advance of surgery that the article on injuries to the chest already stands in need of revision, whilst in that on wounds of joints and bones no mention is made of Bipp. It will be gathered from this short account of the work that it is of extreme value as a supplement to the surgical knowledge of everyone who is called upon to serve in the field or at a military hospital. There is a sufficient index, and the illustrations are good, but the volume weighs 17½ ounces, which is too much for only 189 pages of letterpress.

**The Dietetic Treatment of Diabetes.** By B. D. Basu, Major I.M.B. (retired). Seventh Edition, Revised and Enlarged. (Published by the Panini Office, Bhuvaneshvari Ashram, Bahadurganj. Allahabad. 1916. Price Rs. 1.8.)

In the dietetic treatment of diabetes a difficulty presents itself in the case of persons who are voluntarily vegetarians, or who are prohibited by their religion from eating meat, such as Buddhists and certain castes of Hindoos. In India diabetes is by no means a rare disease; the studies, therefore, of a Hindoo doctor of the dietetic treatment of diabetes in India have peculiar value. Dr. Basu starts with the theory that diabetes mellitus is a manifestation of alimentary toxæmia, and that the increase of sugar in the blood is Nature's antidote to the toxin. The various causes of the presence of sugar in the urine or blood are briefly considered, with the result that Dr. Basu restricts the term "diabetes" to that form of glycosuria which is consequent on alimentary toxæmia.

In India the principal food of the people is wheat and rice, which are both carbo-hydrates. To the potato is attributed by some observers the prevalence of diabetes in India; but our author considers that the increase of the anxiety and worry of life, the use of inferior grains and polished rice for food, and the paucity of milk and ghee are chief causes of alimentary toxæmia.

Dr. Basu asserts that coma occurs most frequently in diabetics who live on flesh foods and take opium; thus he would exclude meat from the dietary of the diabetic, and place him on a vegetarian diet, as that which is best calculated to relieve alimentary toxæmia, and diminish or prevent acidosis.

Spare diet, physical exercise, care of the skin, gastric lavage, rectal douches, and the practice of *Yoga* are recommended. These ancient practices, described in the "Gheranda Samhitâ," are reproduced in modern medicine by the Plombières treatment, abdominal massage, and Swedish drill. In the consideration of dietaries for the diabetic much practical advice is given and some original views put forward. "It is a fact," says Dr. Basu, "that diabetic patients who are vegetarians live longer than those that are meat-eaters," inasmuch as it is the withdrawal, it is explained, of carbo-hydrates from the food which produces auto-intoxication, and leads to acetonuria and finally to coma.

Of carbo-hydrates the following are recommended for diabetics: banana flour, unpolished rice, potatoes, millet, and inulin biscuits; and as substitutes for sugar, honey or acacia gum, and Dr. Basu does not forbid cane-sugar. The book abounds in full quotations and detailed abstracts of the opinions of men of science on the nature and pathology of diabetes, and in accounts of the treatment of the disease by noted physicians of all countries, so that it is a useful compendium of the theories and practice at present in vogue.

**Health of Working Girls.** By Beatrice Webb, M.D., Ch.B. Pp. 103. (London: Blackie and Son, Ltd., 1917. 2s. 6d. net.)

A critic has told how once upon a time he received a book for review: it was, we think, "Treasure Island." Jaded and satiated with the "literature" of the day, he glanced at the book in his office, turned to the beginning and began to read, took it home with him—finding the train journey for once all too short—stood under a lamp-post to finish a chapter, read on during dinner, and sat up until the small hours of the morning to read to the end. In a somewhat similar spirit we have read Dr. Beatrice Webb's little book, every word once at least, and many passages more than once. It is intended as a handbook for welfare supervisors and others. It is to be commended to many others, including nurses and those whose duty it is to study their well-being, as containing twelve remarkably good chapters on personal hygiene for working women. Where all is so good it is difficult to particularise. The best section of the book is perhaps that on anæmia: if we may hint at an omission it must be to regret that a little space is not devoted to advice upon the subject of the working girl's clothing.

We cannot do better than quote some of Dr. Webb's aphorisms:

"The anæmic girl should sleep with her head as nearly on the window-sill as she can get it." (The fond repetition of this phrase may be readily forgiven.)

"Town air is quite good when one gets enough of it."

"Happily the custom of single beds is growing in England."

"The ventilators of trams and omnibuses should be made fool-proof by being fastened permanently open."

"Water is needed in much greater quantity than most women habitually take."

"Feeding is not simply a question of calorie values."

"Most women can eat most things if they go the right way about it."

"By poor food is not necessarily meant cheap food."

"The factory girl is apt to rush out, buy tinned lobster, fried potatoes, pickled cabbage, and a jam tart, and to eat it all in ten minutes . . . with a cup of strong stewed tea."

"Worry is a potent factor in causing indigestion."

"A rest before food is much more important than a rest after food."

"Want of proper work always lands a person, or a part of a person, in serious trouble."

"Pills and salts as commonly taken do nothing to cure constipation . . . proprietors owe their enormous fortunes to the fact that the need for pills recurs perpetually."

"Having teeth filled is almost unknown among the poorer wage-earners."

"Money is better spent on the dentist than on the doctor."

"The front teeth matter little since knives and forks have been invented."

"A tight-laced girl has more headaches than most others."

"For a girl shut up all the week to go to church once on Sunday is as much as she can afford."

"Of all foolish ages for leaving school, and making the plunge into industrial life, fourteen is probably the most foolish."

**The Canteeners.** By Agnes M. Dixon. (London: John Murray. Price 3s. 6d.)

We have here the experiences of a group of ladies who went out to France to administer comforts and coffee to French soldiers. The book is written in a tone of good humour, and friendly insight into the splendid spirit of our Allies. Some good snapshots enliven its pages, and it may be recommended as conveying a cheerful impression of certain creditable activities going on behind the lines.

**Gold Stripes.** By Christie T. Young. (London: Simpkin, Marshall. Price 1s. net.)

This is a breezy and romantic little volume about a Canadian military hospital in England, with which the author is connected in some unindicated way. There is a story of a "lonely soldier," who ran away from home to join his regiment, and refused to go out for fear of meeting acquaintances. At last he thought it less risky to advertise for a cheerful female correspondent. He received 261 replies, some of which are quoted. He answered one because the writer described herself as an Irishwoman, and the lonely soldier was Irish too. The lady arrived; after a decent interval the author looked in and found them hand in hand. She was his mother. This is a sample story from a cheerful little book.

**Bread and Fancy Breads.** By the Honble. Mrs. Lionel Guest. (London: John Lane. Price 6d. net.)

This, another book by the author of "Patriotism and Plenty," is an indispensable possession for every establishment, large or small, where the bread is made at home. Most people will be surprised to find how much can be done to lessen the monotony of meals by improving the baker's art. Biscuits and rusks become no longer expensive luxuries. Griddle-cakes, waffles, muffins, popover, and rolls are well within the reach of every household provided with facilities for baking. And lest any should believe these fancy bakings may lead to extravagance in the use of flour, a large space is devoted to the use of maize flour, buck-wheat flour, bean and bran bread, malt bread, date loaf, and potato scones and cakes. A more valuable budget of recipes has never been offered for sixpence.

